# Supplementary material for: Context-Aware Saliency Guided Radiomics: Application to Prediction of Outcome and HPV-Status from Multi-Center PET/CT Images of Head and Neck Cancer
Source: Cancers (Basel). 2022 Mar 25;14(7):1674. doi: 10.3390/cancers14071674 (PMC8996849; doi:10.3390/cancers14071674)
Supplement: Supplementary file 1 [file cancers-14-01674-s001.zip › cancers-1624248-supplementary.pdf]

Supplementary Materials: Context-Aware Saliency Guided Radiomics: Application to Prediction of Outcome and HPV-Status from Multi-Center PET/CT Images of Head and Neck Cancer

Wenbing Lv, Hui Xu, Xu Han, Hao Zhang, Jianhua Ma, Arman Rahmim and Lijun Lu

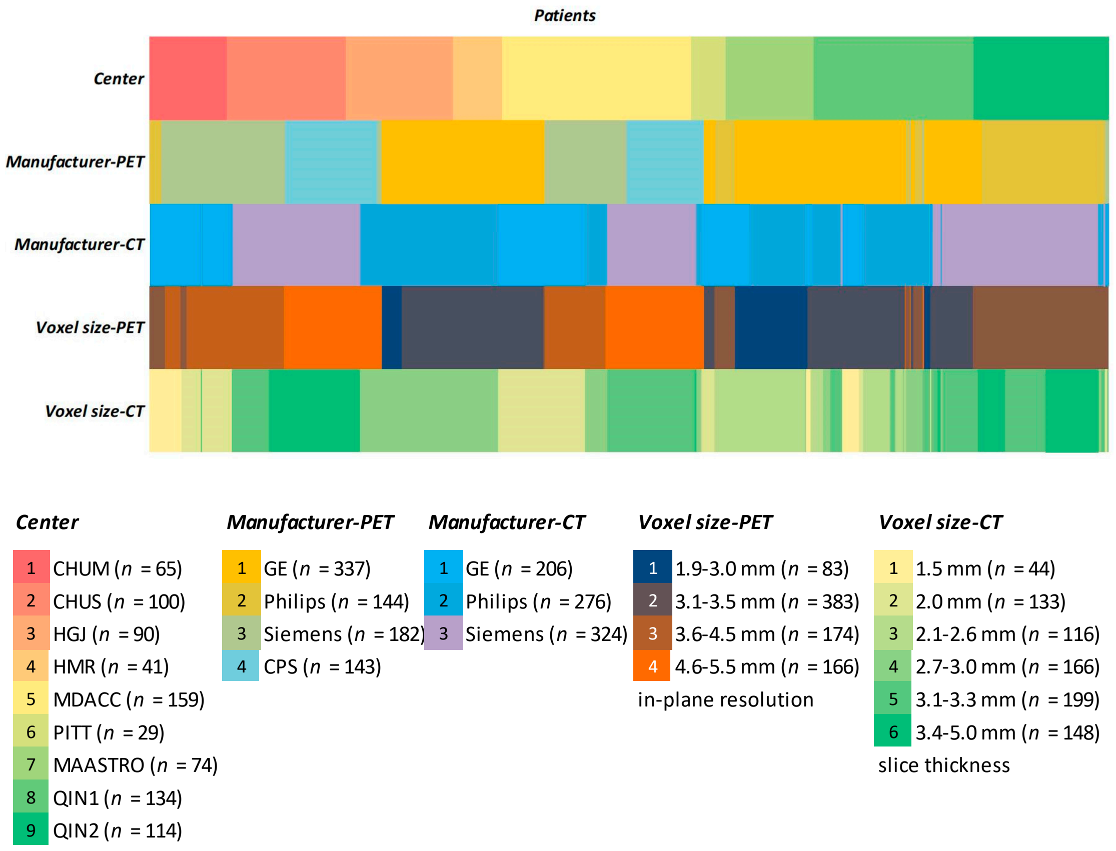

Figure S1. Batches divided by centers, manufacturers and voxel-size. In total, 9, 4, 3, 4, and 6 batches were present for different centers, PET manufacturers, CT manufacturers, PET voxel sizes and CT voxel sizes.

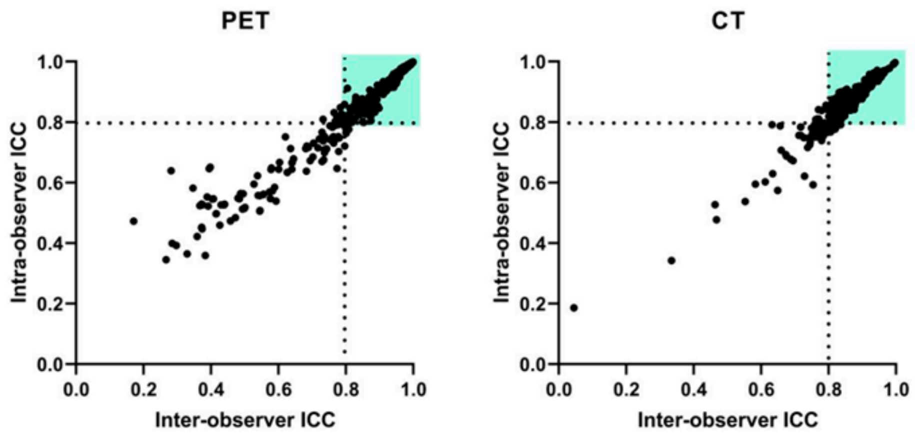

Figure S2. Intra- and inter-observer ICCs of PET and CT features. There were 322/497 PET features and 406/497 CT features with both inter- and intra-observer ICCs>0.8 that remained reproducible for segmentation (green region).

Table S1. C-index (95% confidence interval) of each model (Origin, SalMap, highSal, lowSal, SalxImg, and FusedImg) in training and validation cohorts for RFS, MFS, OS, and DFS predictions.

Model	Training C–index				Validation C–index			
	RFS	MFS	OS	DFS	RFS	MFS	OS	DFS
Origin	0.677 (0.633–0.726)	0.675 (0.623–0.726)	0.644 (0.594–0.695)	0.635 (0.592–0.680)	0.730 (0.652–0.824)	0.719 (0.628–0.812)	0.720 (0.627–0.814)	0.730 (0.653–0.824)
SalMap	0.690 (0.646–0.735)	0.685 (0.633–0.736)	0.661 (0.612–0.710)	0.655 (0.614–0.696)	0.664 (0.584–0.744)	0.712 (0.617–0.808)	0.716 (0.619–0.814)	0.664 (0.586–0.744)
highSal	0.660 (0.614–0.706)	0.638 (0.582–0.690)	0.661 (0.582–0.680)	0.655 (0.585–0.665)	0.664 (0.579–0.769)	0.641 (0.537–0.751)	0.646 (0.541–0.757)	0.664 (0.578–0.766)
lowSal	0.632 (0.579–0.681)	0.661 (0.606–0.716)	0.627 (0.574–0.681)	0.627 (0.581–0.670)	0.701 (0.622–0.800)	0.700 (0.610–0.800)	0.703 (0.612–0.803)	0.702 (0.624–0.801)
SalxImg	0.592 (0.537–0.640)	0.660 (0.606–0.706)	0.645 (0.588–0.694)	0.615 (0.570–0.653)	0.705 (0.623–0.784)	0.760 (0.664–0.859)	0.755 (0.657–0.857)	0.708 (0.628–0.786)
FusedImg	0.604 (0.557–0.655)	0.627 (0.567–0.680)	0.610 (0.554–0.663)	0.595 (0.552–0.639)	0.696 (0.617–0.778)	0.726 (0.628–0.825)	0.726 (0.625–0.827)	0.697 (0.619–0.778)

Table S2. C-index (95% confidence interval) of Rad_Ocm, Rad_HPV, and Rad_Ocm_HPV for outcome prediction in 123 OPC patients from testing cohort, AUC (95% confidence interval) for HPV prediction in 100 OPC patients from training cohort, and 60 OPC patients from external testing cohort.

Model	Rad_Ocm				Rad_HPV						Rad_Ocm_HPV			
	C–index				AUC	AUC	C–index				C–index			
	RFS	MFS	OS	DFS	Training	Testing	RFS	MFS	OS	DFS	RFS	MFS	OS	DFS
Origin	0.502 (0.309–0.667)	0.596 (0.405–0.780)	0.592 (0.458–0.717)	0.564 (0.444–0.676)	0.706 (0.580–0.820)	0.484 (0.278–0.661)	0.588 (0.417–0.759)	0.633 (0.449–0.813)	0.622 (0.476–0.763)	0.615 (0.494–0.740)	0.552 (0.335–0.692)	0.616 (0.417–0.795)	0.611 (0.471–0.728)	0.598 (0.467–0.700)
SalMap	0.592 (0.431–0.733)	0.668 (0.505–0.822)	0.629 (0.503–0.750)	0.613 (0.504–0.719)	0.784 (0.681–0.882)	0.620 (0.480–0.752)	0.554 (0.391–0.719)	0.648 (0.431–0.832)	0.564 (0.431–0.677)	0.544 (0.427–0.658)	0.596 (0.435–0.739)	0.683 (0.498–0.867)	0.603 (0.489–0.738)	0.584 (0.485–0.709)
highSal	0.545 (0.375–0.686)	0.524 (0.335–0.687)	0.509 (0.367–0.624)	0.523 (0.403–0.627)	0.654 (0.521–0.777)	0.670 (0.522–0.820)	0.537 (0.395–0.684)	0.566 (0.395–0.766)	0.578 (0.456–0.696)	0.575 (0.472–0.683)	0.552 (0.393–0.713)	0.588 (0.375–0.759)	0.582 (0.426–0.666)	0.570 (0.446–0.660)
lowSal	0.594 (0.411–0.762)	0.656 (0.472–0.836)	0.562 (0.426–0.683)	0.573 (0.448–0.691)	0.762 (0.634–0.882)	0.555 (0.408–0.695)	0.582 (0.397–0.749)	0.620 (0.437–0.800)	0.597 (0.473–0.707)	0.577 (0.453–0.695)	0.607 (0.420–0.794)	0.683 (0.544–0.839)	0.607 (0.474–0.715)	0.594 (0.471–0.718)
SalxImg	0.546 (0.397–0.683)	0.660 (0.492–0.827)	0.565 (0.433–0.679)	0.567 (0.464–0.666)	0.775 (0.677–0.883)	0.433 (0.263–0.597)	0.641 (0.544–0.749)	0.440 (0.226–0.633)	0.498 (0.375–0.615)	0.512 (0.408–0.618)	0.605 (0.441–0.717)	0.544 (0.397–0.751)	0.534 (0.410–0.657)	0.539 (0.434–0.654)
FusedImg	0.603 (0.460–0.737)	0.638 (0.485–0.784)	0.671 (0.553–0.781)	0.651 (0.546–0.752)	0.702 (0.570–0.831)	0.653 (0.505–0.798)	0.565 (0.458–0.695)	0.687 (0.546–0.853)	0.627 (0.529–0.738)	0.615 (0.533–0.717)	0.616 (0.491–0.731)	0.683 (0.544–0.843)	0.702 (0.599–0.807)	0.684 (0.589–0.780)

Table S3. Features and corresponding coefficient used for Rad_Ocm and Rad_HPV generation in SalMap, SalxImg and FsedImg models. Model configuration (Config.) consists of modality (Clinical, CT, or PET) and harmonization method (e.g. voxel-size based or none).

Model	Config.	Features for Rad_Ocm	Coeff.	Features for Rad_HPV	Coeff.
SalMap	Clin + CT_None	CT_dzm_ldhge_2_5D	0.076	CT_loc_peak_loc	−0.925
		CT_rlm_rl_entr_2D_comb	0.019	CT_cm_corr_2_5D_comb	0.922
		CT_dzm_lde_2_5D	0.183	CT_stat_max	−0.214
		CT_cm_info_corr1_2D_comb	−0.314	T-stage	−0.418
		T-stage	0.156	CT_cm_inv_diff_2_5D_avg	−0.417
		CT_cm_sum_var_2D_avg	0.232	CT_cm_clust_shade_3D_comb	−0.517
		CT_szm_zs_entr_2_5D	−0.041	intercept	1.160
		CT_cm_info_corr2_2D_comb	−0.279		
SalxImg	PET + CT_Voxel size based	PET_szm_glnu_2_5D	0.115	PET_stat_median	−0.385
		CT_ih_min_grad	−0.123	PET_stat_cov	0.089
				PET_ngl_hdhge_3D	−0.380
				PET_cm_joint_max_2_5D_avg	0.018
				PET_stat_skew	0.217
				PET_morph_com	0.708
				CT_cm_info_corr1_2_5D_avg	0.417
				intercept	1.250
FusedImg	PET + CT_Voxel size based	ngl_glnu_2_5D	0.044	cm_info_corr2_3D_avg	0.436
		szm_lzhge_3D	0.070	ngl_hdlge_2_5D	−0.790
		ngl_hdlge_2_5D	0.145	rlm_rl_entr_3D_avg	0.363
				ngl_gl_var_2D	−0.452
				morph_vol_dens_ombb	0.211
				morph_diam	−0.148
				mi_oq	−0.242
				szm_sze_3D	0.182
				loc_peak_glob	−0.009
				rlm_rl_entr_2D_comb	−0.457
				szm_lzlge_2D	0.738
				intercept	1.078
